# A Paper-Based Simulation Model for Teaching Inguinal Hernia Anatomy

**DOI:** 10.1007/s00268-023-07018-0

**Published:** 2023-04-26

**Authors:** Chia Yew Kong, Emma Iddles, Paul Glen

**Affiliations:** Department of General Surgery, Queen Elizabeth University Hospital and School of Medicine, University of Glasgow, 1345 Govan Road, Glasgow, G51 4TF UK

## Abstract

**Background:**

Inguinal hernias remain a challenging area of learning for medical students due to its relatively complex anatomy. Modern curriculum delivery methods are conventionally limited to didactic lectures and demonstration of anatomy intraoperatively. These strategies have limitations; lectures are inherently descriptive and based on 2-dimensional models, while intraoperative teaching is often unstructured and opportunistic.

**Methods:**

A paper-based model was developed comprising three overlapping paper panels simulating the anatomical layers of the inguinal canal which can be modified readily to further simulate various hernia pathologies and their surgical repair. These models were incorporated into a timetabled structured learning session for 3^rd^- and 4^th^-year medical students. Learners responded to fully anonymised surveys before and after the learning session.

**Findings:**

A total of 45 students participated in these sessions over a period of 6 months. Pre-learning session mean ratings for the learners’ confidence in their understanding of the layers of the inguinal canal, identifying indirect and direct inguinal hernias and in naming the contents of the inguinal canal were 2.5, 3.3 and 2.9, while post-learning session mean ratings were 8.0, 9.4 and 8.2, respectively. Paired samples Student’s t-tests for all three questions were statistically significant (*p* < 0.001). The mean rating for usefulness of the session was 9.6/10. Free comments from students emphasised the models’ usefulness as a visual learning aid.

**Discussion and Conclusion:**

Our novel, low-cost paper model was associated with an improvement in learners’ perceived knowledge and understanding of inguinal canal anatomy and pathology.

**Supplementary Information:**

The online version contains supplementary material available at 10.1007/s00268-023-07018-0.

## Introduction

Hernias of the abdominal wall, defined as the abnormal protrusion of intra-abdominal contents through the containing abdominal wall, is a common surgical pathology [1]. They have a prevalence of about 4% in those over 45 years old [2]. Inguinal hernias represent 75% of all abdominal wall hernias, and its repair remains one of the most common general surgical operations in the UK [2, 3].

However, the complex anatomy of the inguinal canal continues to make the understanding of this disease and its surgical repair challenging for medical students and junior surgical trainees [4, 5]. Traditionally, in the undergraduate curriculum, this topic is delivered using didactic lectures and tutorials or delivered in the operating theatre [6]. These modes have inherent limitations; lectures are inherently descriptive and use 2-dimensional images, whereas intraoperative teaching is opportunistic and unstructured.

The COVID-19 pandemic and its subsequent reprioritisation of healthcare resources have consequently led to a detrimental effect on the volume and quality of teaching opportunities in surgical training [7, 8]. This pandemic has highlighted the importance of the development of surgical training tools which can be complementary to traditional surgical training techniques or be used as effective contingency alternatives where normal workplace surgical training is reduced or suspended. This has led to the development and use of a 3D paper-based model for simulated teaching of inguinal hernia in our department.

## Methods

### Hernia model

**Fig. 1:**
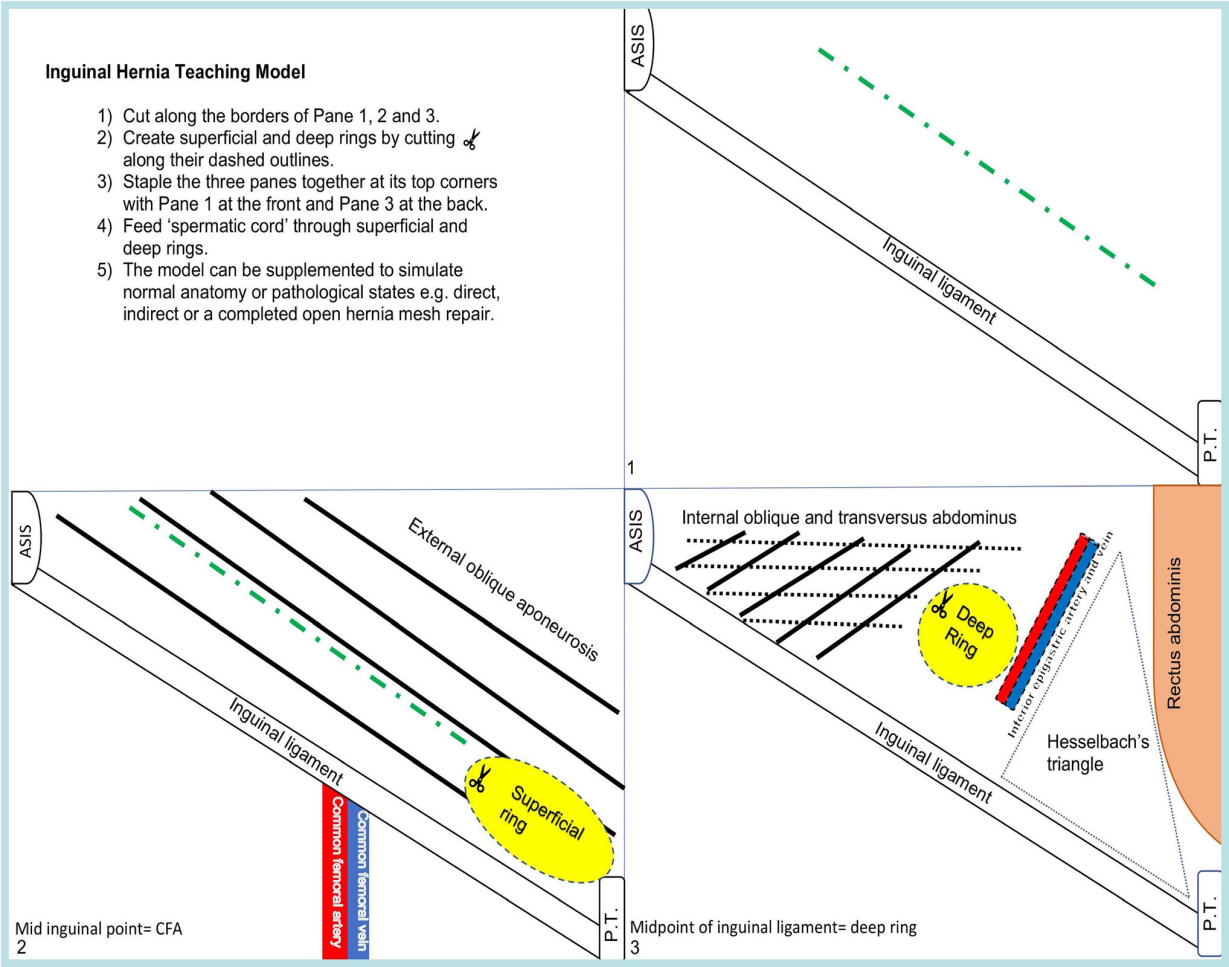
Schematic mage of inguinal canal model comprising four panels (including the instruction panel which is used as the back panel of the model). Sessions utilised these schematic models scaled to A4 size

**Fig. 2:**
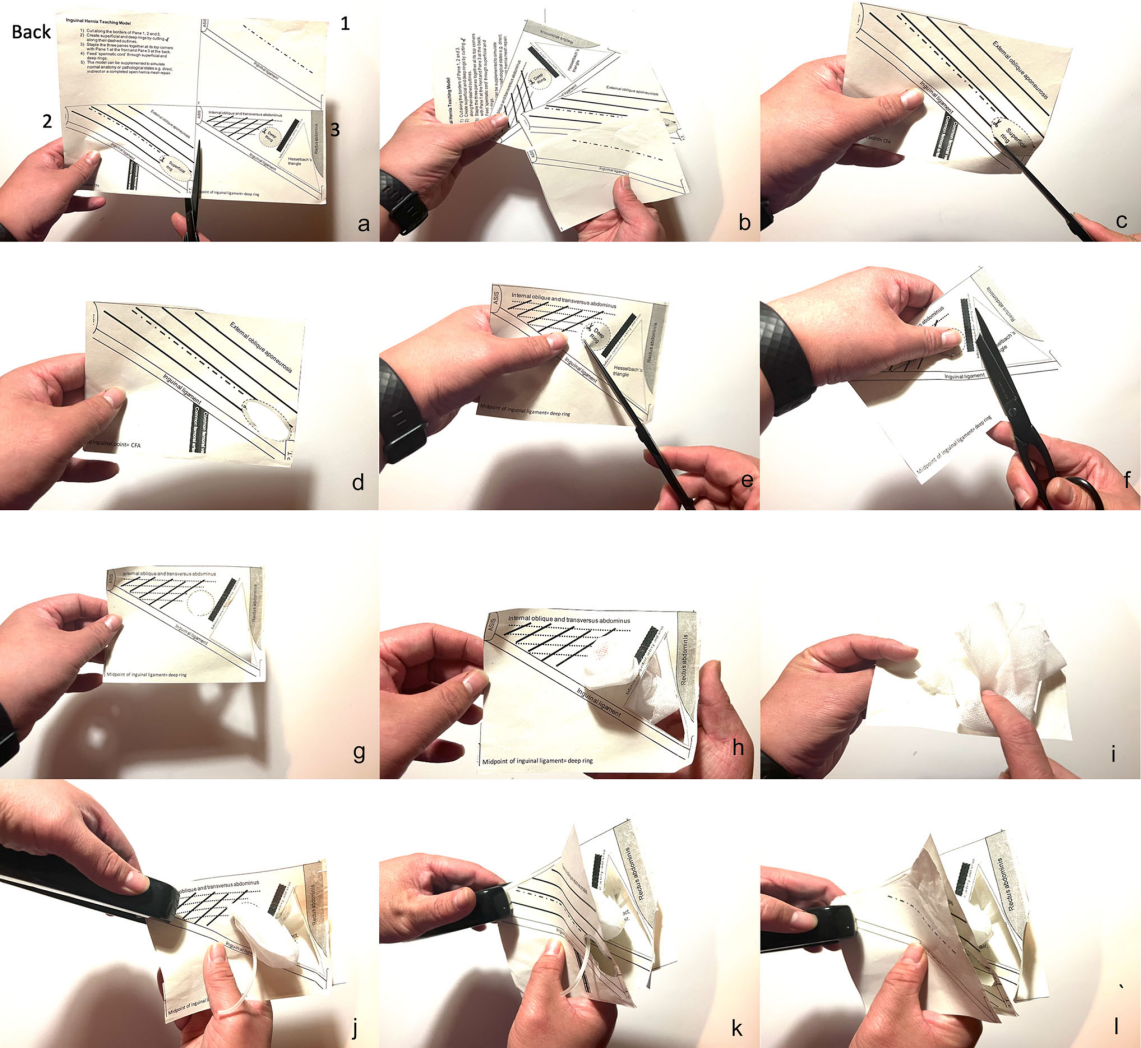
Construction of the inguinal hernia model. **a** and **b** Model scaled to and printed on a A4-sized paper being cut into the 4 individual panels with a pair of scissors. The 4 panels are labelled back (serves as back panel of model and contains instructions on how to construct the model, Panel 1 demonstrating the skin with relevant surface anatomy, Panel 2, the external oblique fascia layer and the inguinal ligament with the superficial inguinal ring as a natural defect in this layer and Panel 3 showing the deep inguinal  ring and its important anatomical relationships. **c** and **d** Superficial ring being cut out from Panel 2. **e**, **f** and **g** Deep inguinal ring being cut out and simulation of weakness in Hesselbach’s triangle by cutting out the area. **h** and **i** Introduction of swabs through the deep inguinal ring in Panel 3 from the front and back, respectively, to simulate an indirect inguinal hernia sac. **j** Stapling of a plastic tube at the top left corner, sandwiched between Panel 3 and the back panel, simulating the spermatic cord or round ligament. The tube is then fed through the deep inguinal ring. **k** Stapling of Panel 2 to the model. The tube is fed through the superficial inguinal ring. **l** Stapling of Panel 1 to the model. Stapling all four corners completes the model

**Fig. 3:**
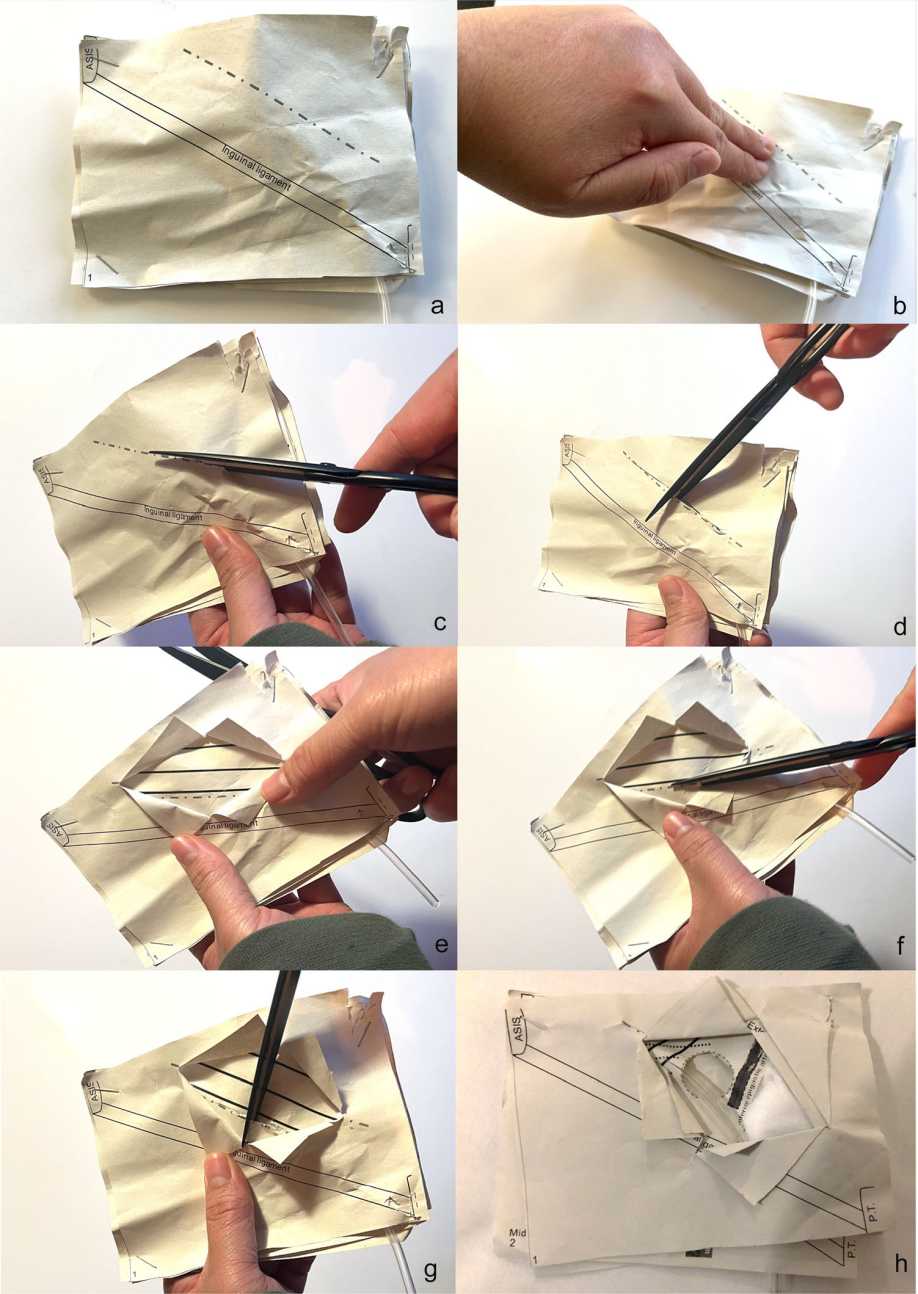
Learners simulating open surgical exposure of the inguinal canal. **a** Model presented to the learners at the start. **b** Model’s ability to allow learners to elicit the clinical sign of a palpable groin lump. **c** The use of scissors to simulate making the initial skin incision at the correct site relative to the relevant surface anatomy. **d** and **e** Learners making perpendicular inferior and superior incisions to the initial skin incision—it is stressed to learners that this is not done in vivo but is done in this model to simulate retraction of tissues for adequate exposure and visualisation of the underlying anatomy. The result of this is the visualisation of the prominent white fibres of the external oblique aponeurosis with fibres going in a parallel direction to the inguinal ligament. **f** and **g** The use of scissors to make an incision in the external oblique aponeurosis to expose the inguinal canal and its contents. Again, it is stressed to learners that this is not done in vivo but is done in this model to simulate retraction of tissues for adequate exposure and visualisation of the underlying anatomy. **h** Fully exposed inguinal canal and evidence of a direct inguinal hernia. Panel 3 of the model is modified so as to simulate multiple different pathologies (see Fig. 4). Learners are then tasked to diagnose the type of hernia from its anatomical relationships fully simulating the initial steps of an open inguinal hernia repair

**Fig. 4:**
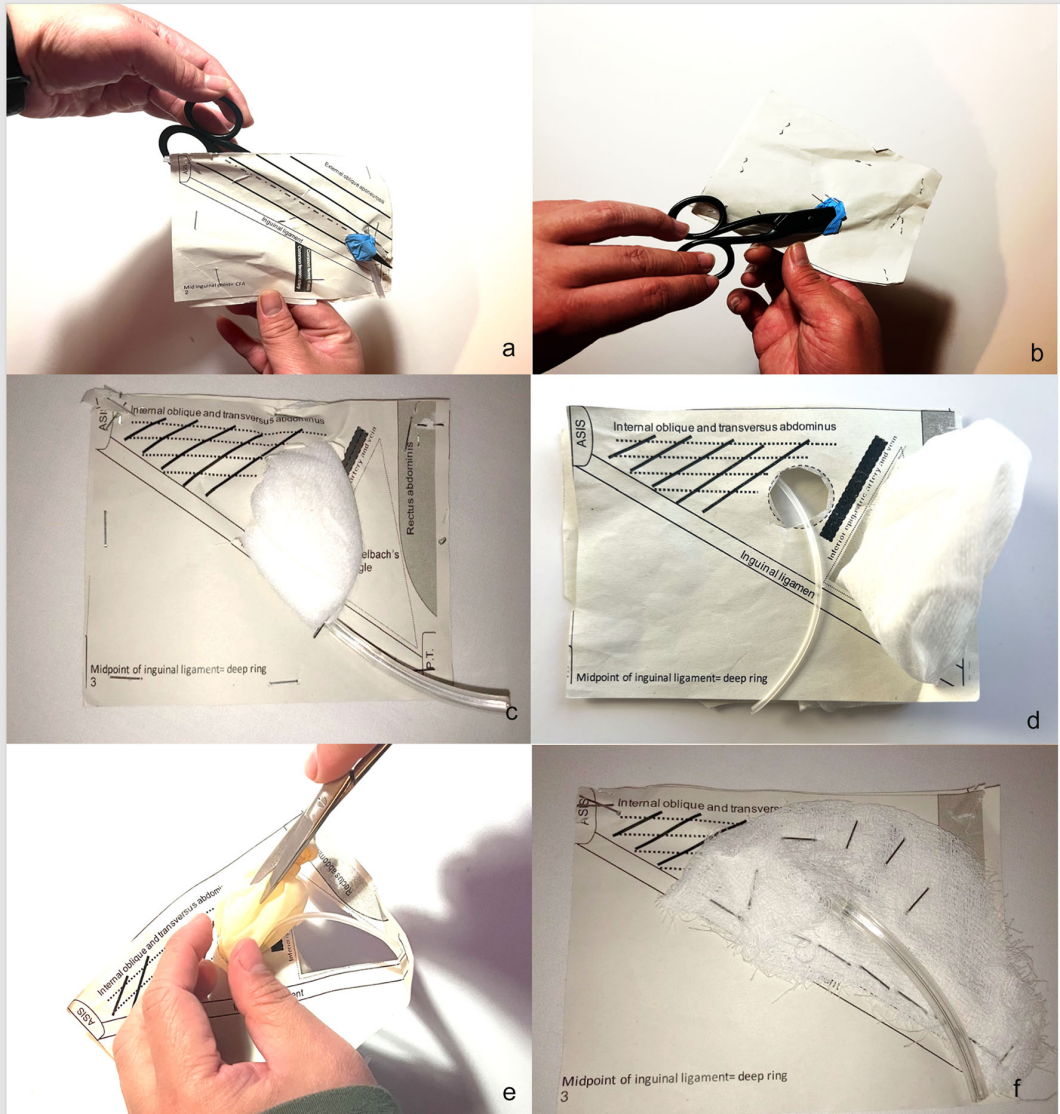
Models in practice. **a** and **b** The use of a pair of scissors to demonstrate anatomical concept of the inguinal canal as an oblique canal  traversing the different layers of the abdominal wall (from the front and back, respectively). The blue material used as a representation of the canal was made from a disposable latex glove. **c** and **d** Different pathologies are simulated using surgical swabs or gauze material, representing an indirect and direct inguinal hernia, respectively. As mentioned, plastic tubing is used to simulate the spermatic cord or round ligament. **e** Simulation of the opening of a hernia sac to visualise and reduce its contents with a disposable latex glove used to simulate the sac. **f** The use of swab to simulate the configuration of a mesh in an open tension-free repair of an inguinal hernia

A paper-based model was developed comprising four overlapping paper panels simulating the anatomical layers of the inguinal canal and associated structures (Figs. 1 and 2). These paper panels display key anatomical structures of the inguinal canal in schematic fashion and allow for low-fidelity simulation of open groin hernia procedures (Figs. 3 and 4).

These models can be easily modified using readily available adjunct materials such as surgical gauze, plastic tubing and glove material to simulate normal inguinal canal anatomy, various inguinal hernia pathologies and an open surgical mesh repair of an inguinal hernia (Figs. 2, 3 and 4).

### Learning sessions

The use of these models was incorporated into a timetabled structured learning session delivered by the authors for 3^rd^- and 4^th^-year medical students rotating through their general surgical placement in a single teaching hospital site.

Briefly, in these learning sessions, pertinent concepts surrounding the anatomy and pathology of inguinal hernia are discussed including surface and surgical anatomy, clinical examination, investigations including radiology, different pathological variants and surgical techniques involved in the repair of inguinal hernia. These learning sessions were designed and blueprinted based on Gagne’s instructional levels [9] (Supplementary Table 1).

Students are then provided with one each of a variety of completed models of the hernia, each constructed to simulate the normal inguinal canal, various inguinal hernia pathologies and a surgically repaired inguinal hernia (Figs. 2 and 4). Students are then allowed to make a ‘skin incision’ on the model and dissect down, simulating a surgical exposure of the inguinal canal in the paper model to the deepest layer and discuss what they find on these models and compare it with the other models (Fig. 3).

In models with simulated pathology, students can proceed to a repair of the hernia, including dissection of the sac and reducing it, and placing and securing the ‘mesh’.

**Fig. 5:**
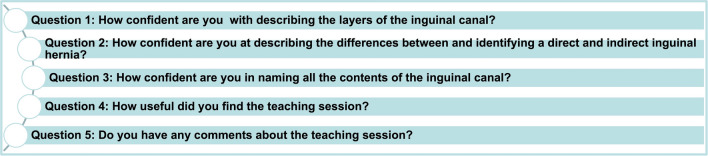
Questionnaire items. Students answered Questions 1–3 pre-learning session; post-learning session students answered Questions 1–3 again as well as Questions 4 and 5

Students’ perceptions of their knowledge and understanding of inguinal hernia anatomy and pathology were assessed using anonymised surveys delivered immediately before and repeated immediately after the learning sessions. Additionally, students’ perceptions of the usefulness of the models and the sessions were assessed in the post-session questionnaires (Fig. 5).

These questionnaires incorporated three questions asking the learners to rate their confidence in describing the layers of the inguinal canal, identifying a direct and indirect inguinal hernia and in naming the contents of the inguinal canal on a 10-point semantic differential scale. Learners were also asked to rate the usefulness of the session and provide freehand comments.

### Ethics

Proportional review has been sought from the University of Glasgow College of Medical, Veterinary and Life Sciences Ethics Committee who have advised that this research project does not need full ethical review and has waived the need for this. Data used and reported in this study are from routinely collected course evaluation data and do not include any personal identifiable details from students involved in these teaching sessions.

## Results

**Fig. 6:**
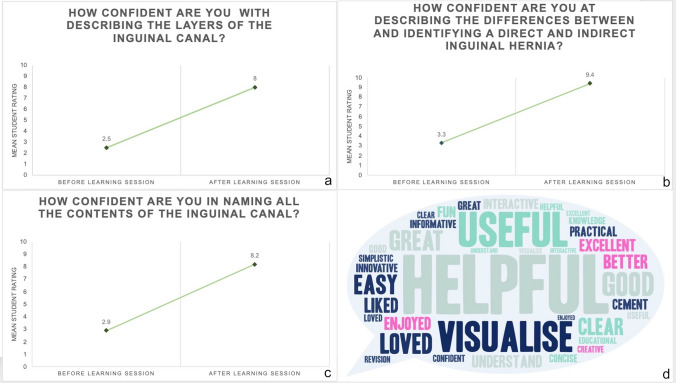
Panels **a**, **b** and **c** Comparative mean student ratings of their confidence on different aspects of inguinal hernia anatomy and pathology using a semantic differential scale from 1 to 10 (1 being not confident at all and 10 being very confident). Panel **d** is a word cloud diagram visualising the verbal feedback received from students via Question 5 asking students for free comments (not to scale)

A total of 45 students participated in these sessions over a period of 6 months. Pre-learning session mean ratings for the learners’ confidence in their understanding of the layers of the inguinal canal, identifying indirect and direct inguinal hernias and in naming the contents of the inguinal canal were 2.5, 3.3 and 2.9, while post-learning session mean ratings were 8.0, 9.4 and 8.2, respectively. Paired samples Student’s *t*-tests for all three questions were statistically significant (*p* < 0.001) (Fig. 6).

The mean rating for usefulness of the session was 9.6/10. Free comments from students emphasised the usefulness of the models as a visual learning aid (Fig. 6).

## Discussion

Our results indicate that students found these sessions useful in improving their understanding of inguinal hernia anatomy, pathology and surgical repair.

Simulation is increasingly used in surgical training and represents a shift from the traditional ‘see one, do one, teach one’ paradigm of surgical training in the past [10]. There have been multiple drivers for this paradigm shift including increasingly steep learning curves associated with modern surgical techniques, an increased focus on patient safety and the adoption of modern educational pedagogical methods in surgical training [10]. Simulation is pedagogically consistent with current understanding of surgical skill acquisition and development. Fitt and Posner describe the three-stage theory of skills acquisition as incorporating the three distinct stages of cognition, integration and automation, which respectively involve intellectualising the task using this and translating it into execution of the task, and thereafter developing automation of the task from continued practice of the task [11]. Simulation allows trainees to develop and master the earliest stages of task acquisition in a safe environment away from the patient.

The evidence for simulated models of hernia repair and their efficacy is scarce in the literature. Ansaloni et al. and Nazari et al. have both independently described different 3-dimensional models constructed from primarily a cardboard box and different fabrics, respectively [4, 12]. Mann et al. describe a full paper model similar to ours illustrated with realistic anatomy [13]. However, unlike our model, Mann et al.’s model does not allow modification for the simulation of different surgical pathologies using adjunct material [13]. Other ex vivo models include computer simulation models have been described but are more cost-intensive and often not available as open-source models which can be reproduced widely by readers and interested trainers [14].

Our model is also considerably low fidelity with the use of paper and schematic anatomy; fidelity in the context of simulation being the level of realism (multidimensional) of a particular simulation activity to the learners [15]. This design is deliberate. Indeed, current evidence suggests that educational outcomes are similar in high- and low-fidelity models and some studies suggest low-fidelity models are superior to high-fidelity models [16, 17]. A more unified interpretation of current evidence may be that training should constitute a range of fidelity levels, and this can be personalised to the individual needs of the learners.

When considering this within the context of cognitive load theory, low fidelity, simpler models may be associated with minimising the intrinsic and extraneous cognitive loads (intrinsic load being the innate difficulty of the task itself; in this case, the hernia repair and extraneous load being any other external loads not related to the subject matter itself, e.g. the learning session and how it is designed) [17]. This can therefore better aid in understanding the key concepts behind the task and therefore acquisition of learning [17]. These suggest that these low-fidelity models are ideally suited towards introducing the concept of hernias and hernia repairs to relative novices such as medical students and surgical trainees at the beginning of training.

Importantly, it is likely that the best learning programs will employ a mixed and perhaps stepwise manner of increasing fidelity and complexity; therefore, our low-fidelity model may be utilised as an introductory level model to introduce the concept to novices before progressing in a stepwise manner to more complex simulations, for example, computer, 3-dimensional, cadaveric and finally patients undergoing hernia repairs in the operating theatre [17, 18].

This work has some limitations. While we have assessed perceptions of knowledge and understanding of medical students, we have not assessed knowledge and understanding levels. Future research should assess  knowledge and understanding levels of students before and after undergoing learning sessions using these models. These models and their efficacy also need to be validated across medical students at different training levels, as well as postgraduate training doctors at the early stages of surgical training.

In conclusion, we describe a cost-effective paper-based model for the teaching of inguinal hernia which can be flexibly modified to represent normal anatomy and different surgical pathologies of the inguinal canal. The use of these models within a structured learning session has been associated with improved students’ perception of their knowledge and understanding of the anatomy, pathology, and management of inguinal hernias.

This paper also provides the model in an electronic template (Supplementary File 2) detailed information on the design and construction of both the model and the associated lesson plans, making this an open-source model which can be evaluated and used by surgical trainers on a global basis.

## Supplementary Information

Below is the link to the electronic supplementary material.

Supplementary file1 (DOCX 16 kb)

Supplementary file2 (PDF 147 kb)
