# Supplementary material for: A Paper-Based Simulation Model for Teaching Inguinal Hernia Anatomy
Source: World J Surg. 2023 Apr 26;47(8):1842–9. doi: 10.1007/s00268-023-07018-0 (PMC10132405; doi:10.1007/s00268-023-07018-0)
Supplement: Supplementary file 1 — Supplementary file1 (DOCX 16 kb) [file 268_2023_7018_MOESM1_ESM.docx]

**Supplementary Table 1:** Lesson plan for the educational session modelled on Gagne’s instructional levels (Gagne RM, Briggs and Wager, 1992)

| **Level** | **Activity** |
| --- | --- |
| 1. Gaining attention | Tell students that we will be using a paper model today to simplify a sometimes-difficult anatomical concept even for junior surgical trainees starting out repair of inguinal hernias. |
| 1. Informing learner of objectives | Students will be made aware of learning outcomes prior to the session and this will be reiterated at the start of the lesson.  1)    Describe the surface, gross and surgical anatomy of the abdominal wall and inguinal canal, including contents of the inguinal canal in males and females  2) Describe the general approach for repairing an inguinal hernia  3)   Differentiate between different types of inguinal hernia  4)    Formulate in brief a plan for investigation and management of inguinal hernia |
| 1. Stimulate recall of prior learning | Ask students whether they have felt hernias or seen a hernia repaired.  Ask about the definition of a hernia.  Ask about types of hernias in the abdominal wall or anywhere else in the body that they know about  Discuss how one would examine a groin lump. |
| 1. Presenting stimulus | Discuss with help of diagrams layers of abdominal wall.  Show anatomical diagrams of the inguinal canal and discuss key anatomy. Clarify that this should become a bit clearer with the paper models.  Ask about different types of inguinal hernia and how this is differentiated. |
| 1. Providing learning guidance | Provide the students with one each of the 4-5 paper hernia models. Tell them we will be simulating a surgical hernia repair today.  Step 1: Orientate students  Ask students to identify what the abbreviations PT and ASIS represents on the models and ask students what laterality/side the hernia model represents.  Step 2: Surgical incision:  Provide them with scissors and get them to cut along dotted line discussing the surface anatomy for placing and making the surgical incision.  Ask students if they know the surface anatomical extent of the inguinal canal and show this on paper model. Ask them about the difference between the mid-inguinal point and midpoint of inguinal ligament. Ask about surface landmark for superficial inguinal ring. Demonstrate all these while students work through models and find these structures on these models.  Step 3: Dissection down to external oblique (2^nd^ layer in model). Elucidate the superficial inguinal ring which has now come into view. Ask students to cut down along dotted line, being careful not to damage structures underneath while doing this, recapitulating a key step in the actual surgical exposure of the inguinal canal.  Step 4: Exposing contents of inguinal canal  Elucidate the visible anatomy now – including deep inguinal ring, inferior epigastric vessels, spermatic cord and the concept of Hasselbach’s triangle.  Show students the extent and oblique nature of the inguinal canal using the paper model.  You may choose to do the next components after working through the models fully including going through the different simulated pathology (elucidated in Step 6: eliciting performance:  Discuss different basic pros and cons of MRI US and CT in hernia investigation (although it is usually a clinical diagnosis).  Ask students to discuss and provide a useful system for remembering contents of inguinal canal in males and females. |
| 1. Eliciting performance | Get students to work through in turn what the model they have represents and their reasoning for it. (I think this is a direct hernia because the hernia sac originates lateral to the inferior epigastric vessels and goes through the deep ring.) Rotate the models through the students.  Show students the general principles of repairing the hernia by repairing a hernia on the simulated model and showing the repaired hernia model. |
| 1. Providing feedback | Continuing real-time feedback is provided throughout   the discussions. |
| 1. Assessing performance | The interactive discussions throughout the session will be a general assessment of the students. |
| 1. Enhancing retention and transfer | Written notes in the form of crib sheets highlighting key concepts are handed out to students. |
