# Supplementary material for: A Paper-Based Simulation Model for Teaching Inguinal Hernia Anatomy
Source: World J Surg. 2023 Apr 26;47(8):1842–9. doi: 10.1007/s00268-023-07018-0 (PMC10132405; doi:10.1007/s00268-023-07018-0)

Inguinal Hernia Teaching Model

1) Cut along the borders of Pane 1, 2 and 3.
2) Create superficial and deep rings by cutting ✂ along their dashed outlines.
3) Staple the three panes together at its top corners with Pane 1 at the front and Pane 3 at the back.
4) Feed ‘spermatic cord’ through superficial and deep rings.
5) The model can be supplemented to simulate normal anatomy or pathological states e.g. direct, indirect or a completed open hernia mesh repair.

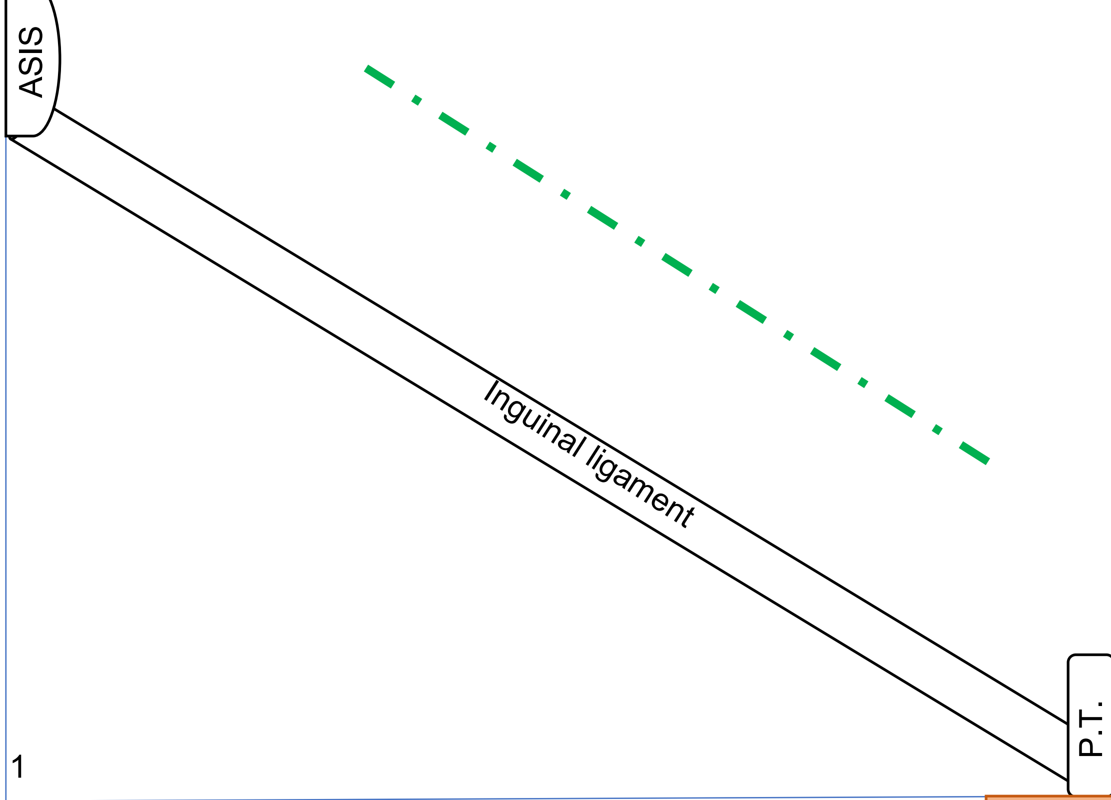

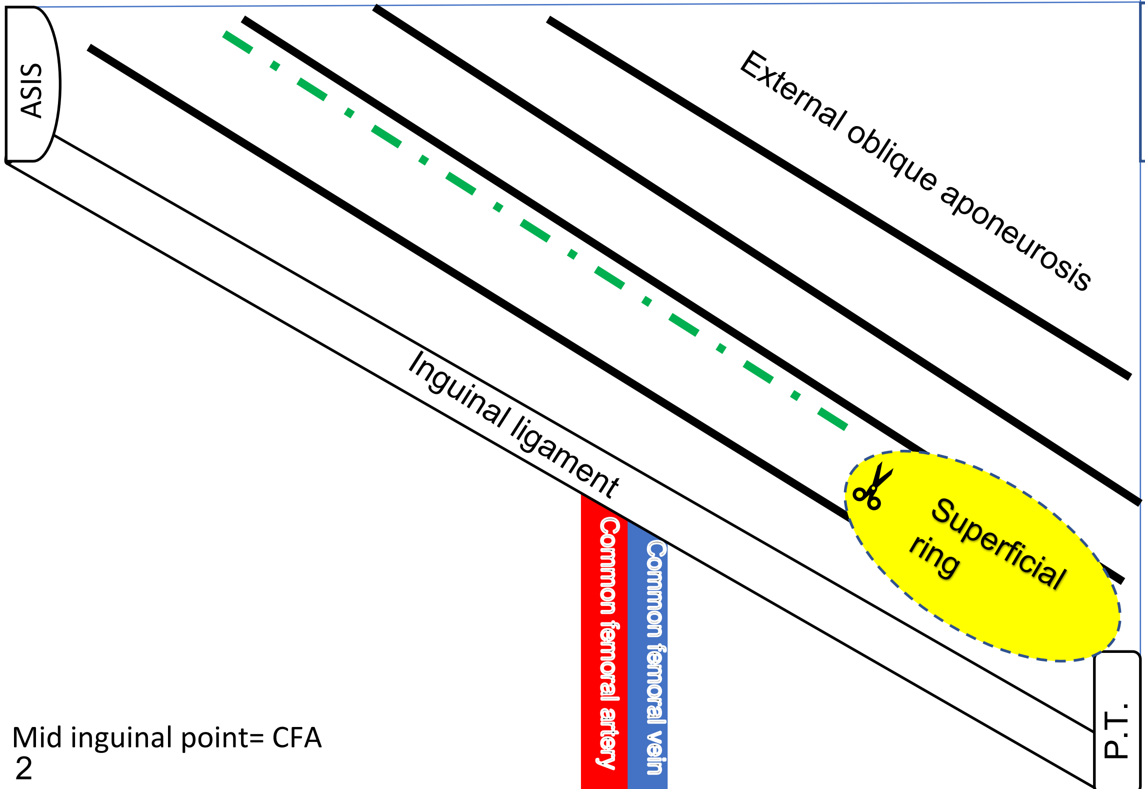

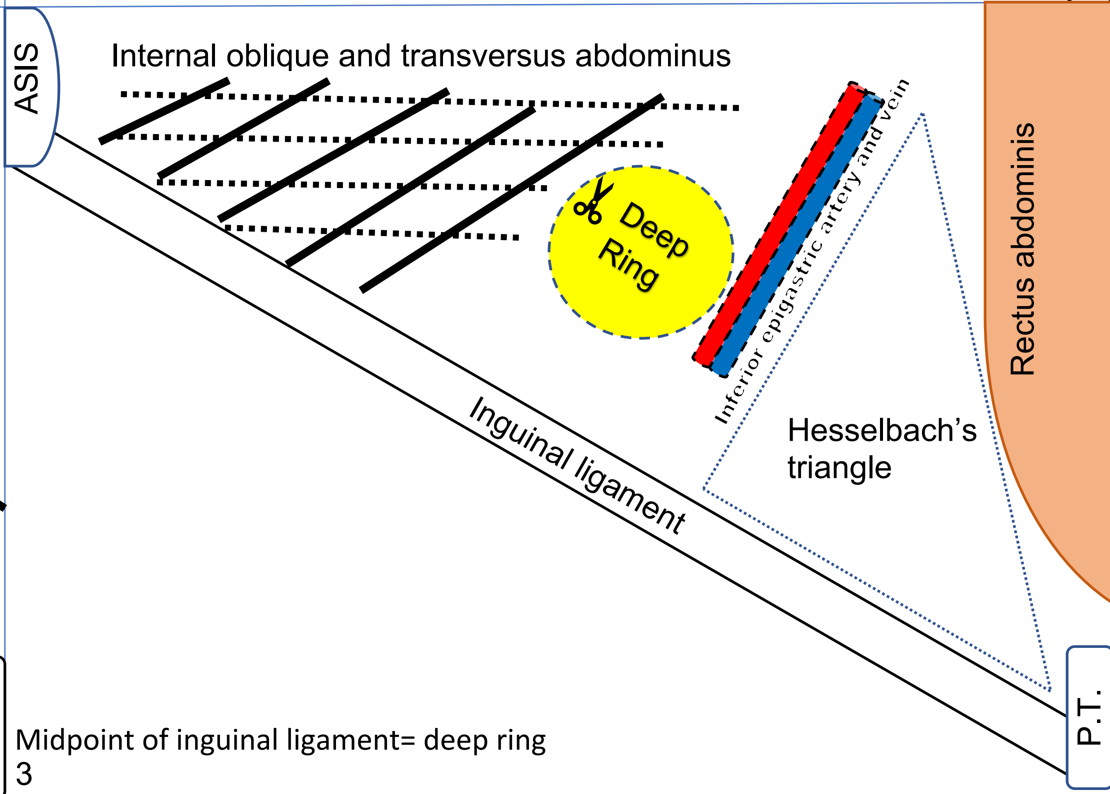

Supplement: Supplementary file 2 — Supplementary file2 (PDF 147 kb) [file 268_2023_7018_MOESM2_ESM.pdf]
